# Isolation of High-Purity Betanin from Red Beet and Elucidation of Its Antioxidant Activity against Peroxynitrite: An in vitro Study

**DOI:** 10.3390/ijms242015411

**Published:** 2023-10-21

**Authors:** Yasuko Sakihama, Takashi Kato, Sopanat Sawatdee, Yo Yakushi, Junichi Asano, Hiroyo Hayashi, Yuya Goto, Makoto Hashimoto, Yasuyuki Hashidoko

**Affiliations:** 1 Division of Applied Bioscience, Research Faculty of Agriculture, Hokkaido University, Kita-9 Nishi-9, Kita-ku, Sapporo 0608589, Japan; 2 Graduate School of Agriculture, Hokkaido University, Kita-9 Nishi-9, Kita-ku, Sapporo 0608589, Japan; 3 School of Energy Science and Engineering, Vidyasirimedhi Institute of Science and Technology, 555 Moo 1 Payupnai, Rayong 21210, Thailand

**Keywords:** peroxynitrite, reactive nitrogen species, betanin, antioxidant, high purity

## Abstract

Reactive oxygen species and reactive nitrogen species (RNS) are damaging for many biomolecules. Peroxynitrite (ONOO^−^) is the most toxic molecular species among RNS. Betalains are known to possess ONOO^−^ scavenging ability. Betanin, a betalain isolated from red beet, possesses antioxidant, anti-inflammatory, and antitumor activities; however, detailed studies of this isolated pigment have not been conducted, owing to its instability under physiological conditions. This study aimed to isolate highly purified betanin from red beetroots using an improved purification method involving deproteinization and citric acid co-precipitation and evaluated its antioxidant activities. The purified betanin thus obtained had a significantly lower isobetanin content than the commercially available betanin dyes. The antioxidant activity of purified betanin examined in the 2,2-diphenyl-1-picrylhydrazyl assay, the direct ONOO^−^ reaction, ONOO^−^-dependent DNA damage, and lipid peroxidation reactions revealed that betanin possessed higher antioxidant capacity than general antioxidants such as ascorbic acid and quercetin. Furthermore, betanin showed indirect and direct cytoprotective effects against H_2_O_2_ and ONOO^−^ cytotoxicity, respectively, in cultured mouse fibroblasts. To the best of our knowledge, this is the first study to demonstrate the cytoprotective effects of betanin against ONOO^−^ toxicity. The highly purified betanin obtained in this study will aid in further exploring its physiological functions.

## 1. Introduction

Reactive oxygen species (ROS) and reactive nitrogen species (RNS) can damage many biomolecules including carbohydrates, lipids, and DNA [1,2], thereby causing many diseases, such as cancer, inflammatory disorders, cardiovascular diseases, and neurodegenerative disorders [3]. Therefore, preventing oxidative stress caused by the accumulation of ROS and RNS is crucial. Peroxynitrite (ONOO^−^) is the most toxic molecular species among RNS. Similar to ROS, it is highly reactive and causes cellular dysfunction by oxidizing and nitrating DNA, proteins, and lipids, which are important cell components.

Antioxidants effectively delay oxidative damage via several mechanisms, such as quenching free radicals directly and stimulating the biosynthesis of enzymatic and non-enzymatic antioxidants [4]. Betalains are water-soluble nitrogenous natural pigments that are found in only 17 families of Caryophyllales; they have received considerable attention due to their antioxidant and anti-inflammatory properties [5]. Betalains possess ONOO^−^ scavenging ability and inhibit DNA damage and the nitrosation of tyrosine in vitro. Betanin, a type of betalain, was isolated from red beet. Red beets are a nutrient-rich superfood, possess anti-inflammatory activity, and exhibit hypotensive, vasodilatory, and hypermotility effects [6,7,8]. The red pigment, betanin, in red beets possesses antioxidant, anti-inflammatory, and antitumor activities [9,10,11]; however, the isolated pigment has not been studied in detail, since it is unstable under physiological conditions [12]. We previously reported the in vitro antioxidant activity of high-performance liquid chromatography (HPLC)-purified betanin from red beet on a small scale against ONOO^−^ [13]. Moreover, the highly pure pigment is not commercially available, which complicates the detailed analysis of its chemical and physiological properties.

Therefore, this study aimed to isolate highly purified betanin from red beetroots using an improved betanin purification method, involving deproteinization and citric acid co-precipitation, and evaluated its antioxidant activities (radical scavenging, DNA cleavage prevention, and lipid peroxidation inhibition activities). In addition, its cytoprotective activity against ONOO^−^ toxicity was also examined using the NIH3T3 fibroblast cell line.

## 2. Results

### 2.1. Purification of Betanin

Since betanin is unstable and prone to decomposition during processing, refining, and storage, it is difficult to isolate high-purity betanin. Hence, commercial betanin dyes contain large amounts of stabilizers, such as dextrin. The amount of betanin per powder weight of both commercial and purified betanin was determined by measuring the absorbance at 538 nm. The findings revealed that the betanin content of the commercial betanin powder was very low, being less than 1% (Figure S1A). In contrast, the betanin content of the betanin powder purified in this study was more than 95% (Figure S1B).

**Figure 1:**
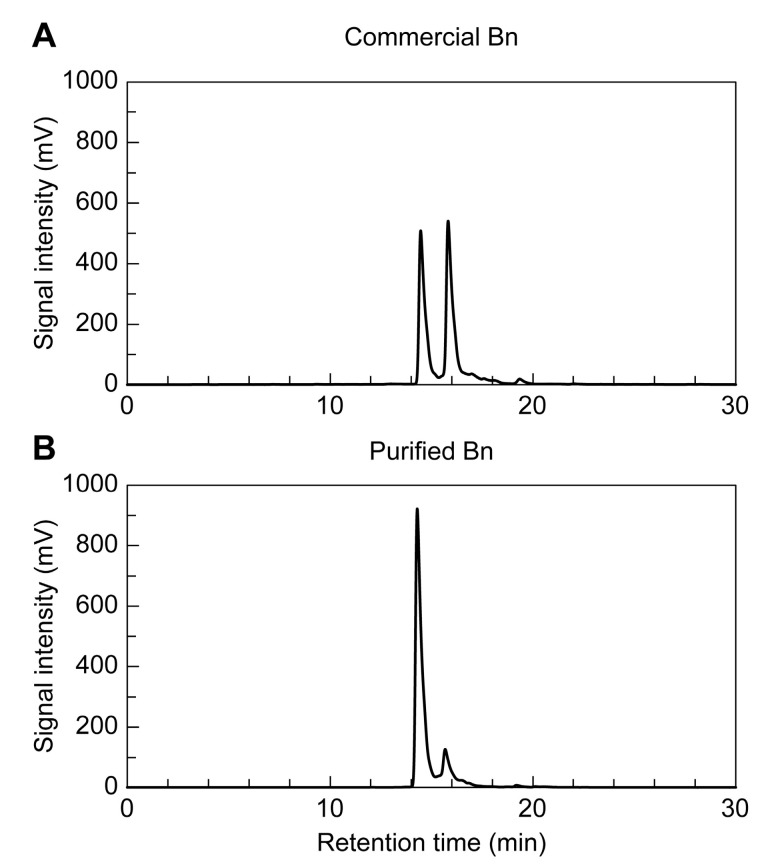
High-performance liquid chromatography (HPLC) analysis. (**A**) Commercial betanin (Bn) at 1 mM, (**B**) purified Bn at 1 mM. Peaks represent betanin and isobetanin.

HPLC analysis of commercial betanin and betanin purified using the method developed in this study revealed betanin and isobetanin peaks in a 1:1 ratio in commercial betanin (Figure 1A), whereas the purified betanin contained a much lower percentage of isobetanin and more than 90% of betanin (Figure 1B). Both commercial and purified betanin were monitored at 280 nm, and small peaks were detected at the retention times of 14.6 min and 16.0 min (Figure S2). These were considered to be derived from betanin and isobetanin based on the retention times and relative signal intensities. As evident from the absorption results at 280 nm, purified betanin appeared to contain fewer foreign substances, such as proteins and phenolic compounds, compared to commercial betanin. The betanin powder purified using the developed method was stable under refrigeration for at least 2 years.

### 2.2. Antioxidative Activity of Betanins, Quercetin, and Ascorbic Acid

The total antioxidant activity evaluated using the 2,2-diphenyl-1-picrylhydrazyl (DPPH) assay was expressed in terms of DPPH IC_50_ and Trolox equivalent antioxidant activity (TEAC) values (Table 1). Although the DPPH IC_50_ values of all betanin compounds showed insignificant differences, these compounds showed significantly higher antioxidant activity than standard antioxidants such as quercetin, ascorbic acid, and Trolox. Among the betanin compounds, commercial betanin had the highest antioxidant activity. The antioxidant capacity of the analytical samples followed the order: commercial betanin > purified betanin > quercetin > ascorbic acid > Trolox.

### 2.3. Direct Reaction of ONOO− with Purified Betanin

**Figure 2:**
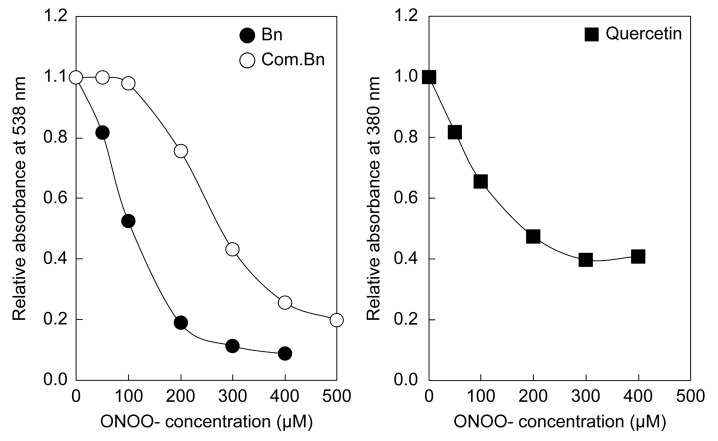
Changes in the absorbance of betanin and quercetin post ONOO^−^ addition. Purified betanin (Bn), commercial betanin (Com. Bn), and quercetin were reacted with increasing concentrations of ONOO^−^. The decrease in absorbance depended on the concentration of ONOO^−^. Each value is expressed as a relative value, with the absorbance without ONOO^−^ indicated as 1.0.

The analyzed compounds were directly reacted with ONOO^−^ to determine their ONOO^−^ scavenging activity. Betanin or quercetin were added in phosphate buffer (pH 7.2), and different concentrations of ONOO^−^ were added. The absorbance was measured after 3 min (Figure 2). The absorbance of quercetin decreased with an increase in the concentration of ONOO^−^ and stabilized at concentrations above 300 µM. Similarly, the absorbance of purified and commercial betanin also showed a decrease with an increasing ONOO^−^ concentration, and the a plateau was reached above 300 µM and 500 µM ONOO^−^, respectively. Evaluation of ONOO^−^ reactivity per µM of each compound revealed that quercetin, purified betanin, and commercial betanin reacted with approximately 15, 50, and 72 µM ONOO^−^, respectively, suggesting increased reactivity with ONOO^−^ for both betanins compared to quercetin. Furthermore, unlike quercetin and purified betanin, commercial betanin did not show a decrease in absorbance at ONOO^−^ concentrations below 100 µM, suggesting that it was less reactive at low ONOO^−^ concentrations.

### 2.4. Inhibition of ONOO^−^-Dependent Plasmid DNA Cleavage by Purified Betanin

**Figure 3:**
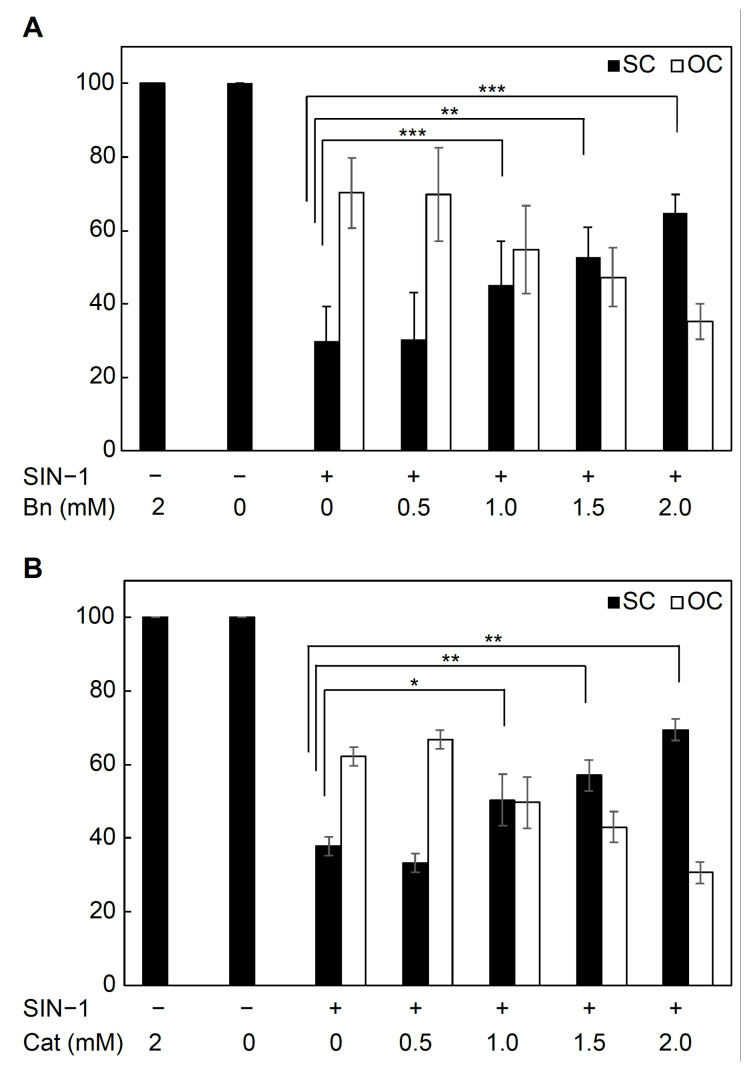
The DNA damage protective ability of (**A**) purified betanin and (**B**) catechin. The average of quantified band intensity for supercoiled (SC) and open circular (OC) forms was estimated using the ImageJ software. Each experimental value is expressed as mean ± standard deviation (*n* = 3 for purified betanin, *n* = 4 for catechin). Statistical significance is indicated by * *p* < 0.05, ** *p* < 0.01, *** *p* < 0.001 (Dunnett’s test).

The protection conferred by betanin against DNA damage was investigated by incubating the pUC19 plasmid DNA in 0.1 mM 3-(4-morpholinyl) sydnonimine hydrochloride (SIN-1), an ONOO^−^ generator. The treatment of supercoiled (SC) pUC19 plasmids with SIN-1 converted approximately 60–70% of pUC19 plasmids to single-strand-break open circular (OC) forms. In the presence of 0.5–2.0 mM betanin, the abundance of OC plasmids decreased, and that of SC plasmids increased in a betanin concentration-dependent manner (Figure 3A). Similar results were obtained when catechin was added instead of betanin (Figure 3B). The plasmid protection abilities of betanin and catechin were almost equivalent.

### 2.5. Inhibition of ONOO^−^-Dependent Lipid Peroxidation by Purified Betanin

**Figure 4:**
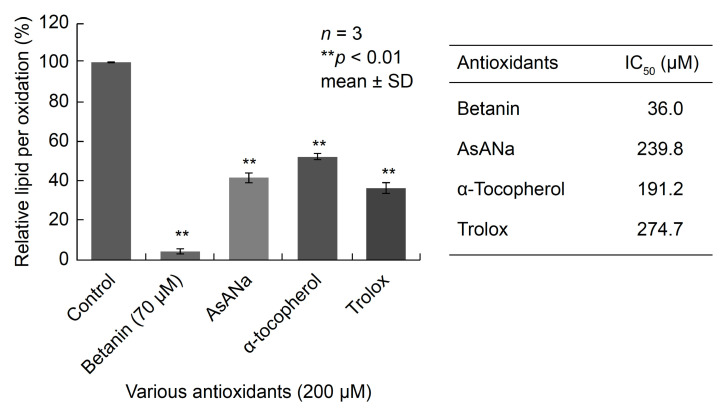
Inhibition of liposome oxidation by betanin and other antioxidants. Each experimental value is expressed as mean ± standard deviation (*n* = 3). Statistical significance is indicated by ** *p* < 0.01 (Dunnett’s test). The TBARS IC_50_ value indicates the concentration required to halve the amount of thiobarbituric acid reactive substances induced by the addition of 1 mM ONOO^−^. AsANa: ascorbic acid sodium salt.

A liposome solution without ONOO^−^ containing 0.6 nmol of thiobarbituric acid reactive substances (TBARSs) was used. A TBARS calibration curve was prepared using malondialdehyde (MDA) (Figure S3A). The addition of 0.5, 1, and 1.5 mM ONOO^−^ to the solution resulted in a 2-, 3-, and 3.8-fold increase in TBARS content, respectively, indicating that lipid peroxidation was dependent on ONOO^−^ concentration (Figure S3B). The increase in TBARS content by the addition of 1 mM ONOO^−^ was considered as 100%, and the remaining data for lipid peroxide formation are presented as relative values (Figure S3C). The findings revealed that the 1 mM ONOO^−^-induced increase in TBARSs remained unchanged in the presence of 10 µM betanin and decreased by 47% and 28% on adding 30 µM and 50 µM betanin, respectively. The addition of 70 µM betanin significantly suppressed TBARS formation, leading to a level that was less than 5% of that without betanin (Figure 4). Comparing the concentrations of the examined compounds required to inhibit ONOO^−^-dependent lipid peroxidation by 50% (TBARS IC_50_), the IC_50_ of betanin (36.0 µM) was considerably lower than those of the other antioxidants ascorbic acid, α-tocopherol, and Trolox, which were 239.8, 191.2, and 274.7 µM, respectively.

### 2.6. Stability of Purified Betanin under Cell Culture Conditions

**Figure 5:**
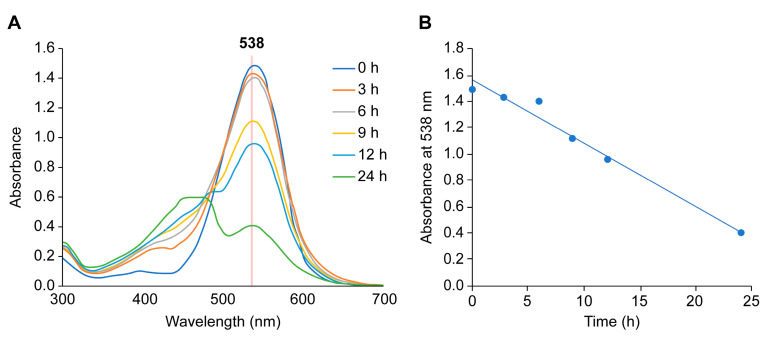
Degradation of betanin under cell culture conditions. Purified betanin (25 μM) was incubated in Dulbecco’s modified Eagle medium containing fetal bovine serum and penicillin/streptomycin at 5% CO_2_, 37 °C. The absorbance was also measured using betanin-free cell culture medium as a control. (**A**) Ultraviolet–visible spectra over time, (**B**) absorbance at 538 nm over time.

A spectroscopic analysis of an NIH3T3 fibroblast cell culture supernatant with 25 µM betanin revealed changes in the absorption spectrum with time (Figure 5A). In particular, the absorbance at 538 nm, the maximum absorption wavelength of betanin, decreased over time to approximately 90, 60, and 25% at 3, 12, and 24 h (Figure 5B), respectively, while the absorbance at 450 nm increased with time. These data suggested that, along with betanin, its degradation products should also be considered during a long-term betanin treatment. The decrease in absorbance at 538 nm with time was unaffected by the presence or absence of cells. Therefore, it is suggested that betanin is spontaneously degraded under cell culture conditions.

### 2.7. Betanin-Induced Protection against H_2_O_2_

**Figure 6:**
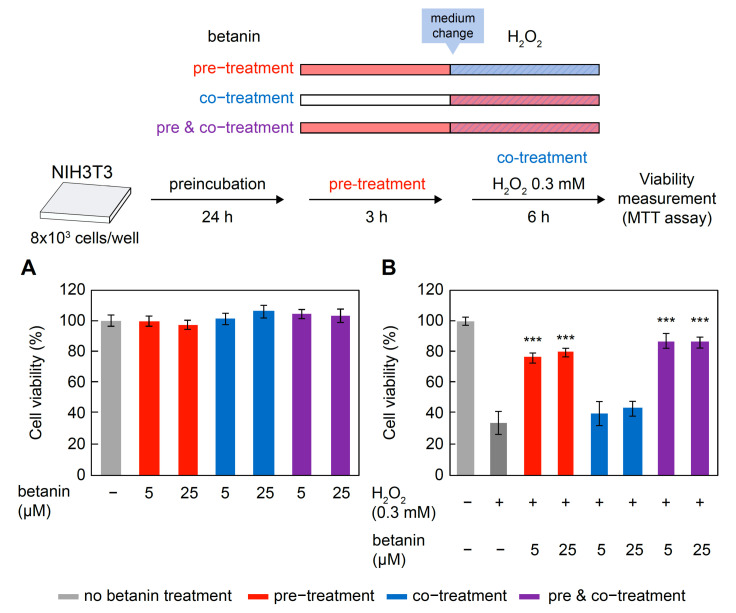
Effect of 3 h betanin pre-treatment and betanin co-treatment on H_2_O_2_ cytotoxicity. Cell viability is expressed as a relative value with respect to the control (untreated) at 100%. (**A**) Cells were treated with betanin only, (**B**) cells were treated with betanin and H_2_O_2_. Data are expressed as mean ± standard error of the mean (*n* = 3). Data were statistically assessed using Dunnett’s test, and significance is indicated as *** *p* < 0.001 compared with the H_2_O_2_ treatment group.

H_2_O_2_ toxicity in NIH3T3 cells and the cytoprotective effect of betanin were examined. Betanin was found to be non-cytotoxic at concentrations below 25 μM in the 3 h, 6 h, and 3 h pretreatment + 6 h co-treatment groups (Figure 6A). Treatment with 0.3 mM H_2_O_2_ for 6 h decreased cell viability to approximately 40% relative to the untreated group. However, cell viability significantly increased to approximately 80% of that of the untreated group (*p* < 0.001) in the 3 h betanin pre-treatment and 3 h pre-treatment + 6 h co-treatment groups, compared to that of the group treated with H_2_O_2_ alone (Figure 6B). In contrast, no increase in cell viability was observed on co-treatment with betanin and H_2_O_2_, compared to that of the group treated with H_2_O_2_ alone (Figure 6B). This suggests that betanin at 5 µM and 25 µM concentrations did not exert direct cytoprotective effects against H_2_O_2_ in the co-treatment groups but exerted indirect cytoprotection during pre-treatment for 3 h. In addition, cell viability after 24 h of betanin pre-treatment was significantly higher than those measured in the presence of H_2_O_2_ alone (*p* < 0.001) and after a 3 h pretreatment (Figure S4).

### 2.8. Effect of Betanin Treatment on Nuclear Factor-Erythroid 2-Related Factor 2 (NRF2) Target Gene Expression

**Figure 7:**
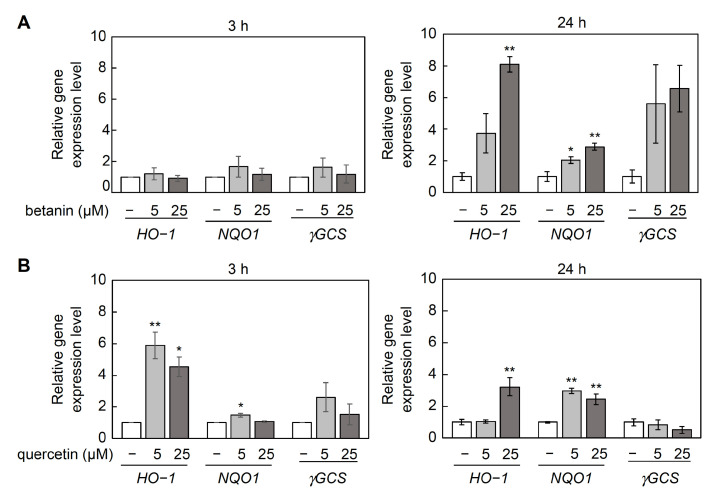
Effect of betanin or quercetin treatment for 3 h and 24 h on Nrf2 target gene expression. Relative gene expression levels were quantified using real-time quantitative polymerase chain reaction in NIH3T3 cells treated with (**A**) betanin (5, 25 µM) or (**B**) quercetin (5, 25 µM) for 24 h. Data are expressed as mean ± standard error of the mean (*n* = 3). Data were statistically assessed using one-way analysis of variance, followed by Dunnett’s test; significant values are indicated by * *p* < 0.05, ** *p* < 0.01 compared with the non-treatment group (control). *HO-1*: Heme oxygenase-1, *NQO1:* NAD(P)H quinone oxidoreductase 1, *γGCS*: γ-glutamylcysteine synthetase (reference gene: GAPDH).

Nrf2 is a transcription factor that induces antioxidant defense mechanisms in response to oxidative stress [14,15]. Betanin treatment for 72 h induces the expression of antioxidant-related genes regulated by Nrf2 [16]. Therefore, NIH3T3 cells were treated with betanin (5, 25 µM) or quercetin (5, 25 µM) for 3 or 24 h, and the effect on Nrf2 target gene expression was evaluated using real-time quantitative polymerase chain reaction (Figure 7). Treatment with betanin for 24 h, but not for 3 h, significantly induced the expression of Nrf2-regulated antioxidant genes such as heme oxygenase-1 *(HO-1)* and NAD(P)H quinone oxidoreductase (*NQO1*) (Figure 7A). However, both 3 h and 24 h treatments with quercetin significantly induced the expression of *HO-1* and *NQO1* (Figure 7B).

### 2.9. Evaluation of the Cytoprotective Effects of Betanin on Peroxynitrite

**Figure 8:**
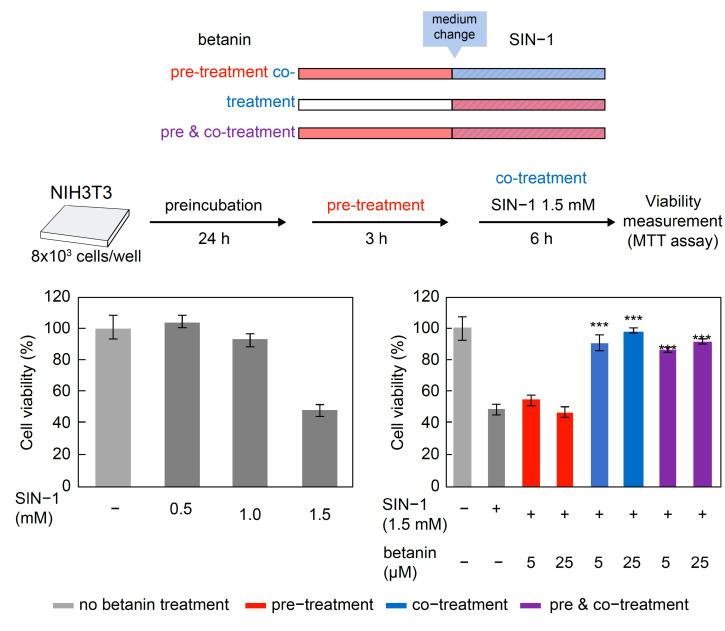
Effect of 3 h betanin pre-treatment and betanin co-treatment on SIN-1 cytotoxicity. Cell viability is expressed as a relative value with respect to the control (untreated) at 100%. Data are expressed as mean ± standard error of the mean (*n* = 3). Data were statistically assessed using Dunnett’s test, and significance is indicated as *** *p* < 0.001 compared with the SIN-1 treatment group.

The effects of betanin or quercetin on the cytotoxicity of the ONOO^-^ donor SIN-1 were determined. Cell viability was measured after 3 h of betanin treatment followed by SIN-1 treatment. The results showed that cell viability decreased in a concentration-dependent manner in the presence of SIN-1 alone at a concentration between 0.5 and 1.5 mM; cell viability decreased by approximately 50% after 6 h of treatment with 1.5 mM SIN-1 (Figure 8, left). Therefore, 1.5 mM SIN-1 was used for the subsequent treatments. Cell viability significantly increased (*p* < 0.001) with betanin co-treatment compared to that measured after treatment with SIN-1 alone (Figure 8, right). In contrast, cell viability did not increase on pre-treatment with betanin for 3 h, compared to that measured in the presence of SIN-1 alone.

Since the simultaneous treatment of betanin or quercetin with SIN-1 inhibited SIN-1-induced cell death, it is suggested that betanin or quercetin inhibited the accumulation of intracellular ROS/RNS induced by SIN-1 treatment. Therefore, we attempted to detect the intracellular ROS/RNS accumulation using 2’,7’-dichlorodihydrofluorescein diacetate (DCFH-DA).

**Figure 9:**
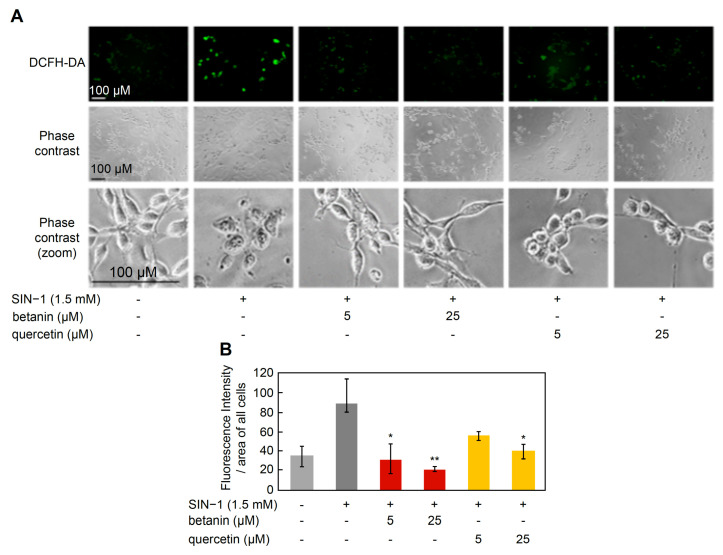
Intracellular ONOO^−^ detection. (**A**) Fluorescence microscopy images. (**B**) Fluorescence intensity per cell area. Scale bars, 100 μm. Data were statistically assessed using Dunnett’s test, and significance is indicated as * *p* < 0.05, ** *p* < 0.01 compared with the SIN-1 treatment group.

After co-treatment of NIH3T3 cells with betanin or quercetin (5, 25 µM) and SIN-1 for 6 h, cell-accumulated ROS/RNS were detected fluorescently using DCFH-DA. Cell morphology and DCF fluorescence were observed using a fluorescence microscope. DCF fluorescence was observed in cells treated with SIN-1 alone and was found to decrease on co-treatment with betanin and quercetin (Figure 9A,B). In particular, DCF fluorescence intensity was significantly reduced to the control (untreated) level at 5 µM and 25 µM betanin concentrations, compared to that measured in the presence of SIN-1 alone (* *p* < 0.05, ** *p* < 0.01). In contrast, DCF fluorescence intensity significantly decreased in the presence of 25 µM quercetin (* *p* < 0.05), compared to that measured in the presence of SIN-1 alone, and a decreasing trend in fluorescence intensity was observed at 5 µM. In addition, phase-contrast observation of cell morphology revealed many cells with retracted pseudopodia in the SIN-1-alone treatment group (Figure 9B). A simultaneous treatment with betanin alleviated the tendency of pseudopodia retraction, and the morphology of the pseudopodia was similar to that of the control (non-treated) cells. In contrast, more pseudopodia were observed in the quercetin-treated cells than in the control (untreated area) and betanin-treated cells (Figure 9A). Thus, betanin appeared to possess a stronger ONOO^−^ scavenging ability than quercetin.

## 3. Discussion

In this study, we were able to efficiently purify betanin in powdered form by deproteinization and citric acid co-precipitation. A HPLC analysis revealed that commercial betanin contained betanin and its isomer, isobetanin, in a 1:1 ratio, whereas betanin purified by our method had a much lower content (<10%) of isobetanin and low isomerization. In addition, the betanin powder purified in this study was stable for more than 2 years at 4 °C and in the dark, according to HPLC analysis. Betanin is usually unstable in solution and relatively stable in the solid state. The underlying reason for purified betanin being stable for more than 2 years is its crystallization. Commercial betanin is also thought to be stable for several years in the solid state, but it has not yet been confirmed whether it is stable after the removal of dextrin, which constitutes more than 95% of commercial betanin. 

Further comprehensive investigation is needed to elucidate the stability of betanin. It is known that ascorbic acid reacts with dissolved oxygen in solution, leading to the generation of H_2_O_2_. Furthermore, the coexistence of transition metal ions like copper and iron with ascorbic acid and other redox-active compounds may potentially accelerate the production of H_2_O_2_ and the degradation of ascorbic acid [17,18]. Additionally, Grzesik et al. reported the production of H_2_O_2_ in the cell culture medium D-MEM, which was also used in this experiment, due to polyphenols such as propyl gallate, (-)-epigallocatechin gallate, and quercetin [19]. In consideration of the capacity of polyphenols to generate H_2_O_2_ in cell culture medium, Long and colleagues recommend distinguishing between the direct intracellular effects of phenolic compounds and the effects resulting from H_2_O_2_ generated in the culture medium. They proposed investigating the behavior of added phenolic compounds, quantifying H_2_O_2_, and evaluating the impact of catalase supplementation [20]. In this study, betanin exhibited antioxidative activity comparable to or even stronger than those of ascorbic acid and flavonoids. This suggests the potential for betanin to generate H_2_O_2_ in cell culture media, such as D-MEM, similar to ascorbic acid and polyphenols, and it cannot be ruled out that betanin-dependent H_2_O_2_ generation might have influenced the stability of betanin under the cell culture conditions shown in Figure 5. Therefore, for future research on the intracellular effects of betanin, quantitative measurements of H_2_O_2_ generation in the culture medium during betanin treatment and the analysis of the degradation products of betanin could provide new insights into the mechanisms of betanin instability.

Betanin was shown to protect cells from H_2_O_2_-induced cell death even after a short pretreatment of 3 h. Additionally, pretreatment with betanin for 24 h significantly induced Nrf2-regulated antioxidant gene expression, a result similar to that previously reported after a 72 h pretreatment [16]. Furthermore, the 3 h betanin pretreatment showed significant cytoprotection comparable to that induced by the 24 h pretreatment; however, no significant induction of Nrf2-regulated antioxidant genes was observed with the 3 h pretreatment. These results suggest that betanin might have exerted its cytoprotective action through different mechanisms in cells pretreated for 3 h. Furthermore, as shown in Figure 5, more than 90% of betanin remained undegraded after 3 h at 37 °C, which were the cell viability assay conditions, and all assays other than the cell viability assay performed in this study were performed within 1 h at room temperature or 37 °C (DPPH assay: 30 min; direct reaction: 3 min; TBA assay: 5 min; DNA cleavage assay: 37 °C, 1 h); so, the effect of betanin degradation was considered minute.

The inhibition of H_2_O_2_-induced DNA damage by betanin or a betalain-rich Cactus pear (*Opuntia ficus-indica*) fruit extract was reported in several human cell lines, such as human peripheral lymphocytes and the colon carcinoma cell line HT-29. In these studies, a 14–72 h betanin treatment showed protective effects against H_2_O_2_ toxicity [8,16,21,22]. However, there are still some problems related to betanin use, such as its low purification level and the fact that its stability during long-term administration has not been investigated. In addition, the reactivity of betanin with active nitrogen has not been evaluated at the cellular level. Therefore, we evaluated the cytoprotective effect of purified betanin against ROS/RNS at a treatment time when betanin was not degraded.

Betanin was found to be highly unstable under cell culture conditions, with an approximately 50% reduction in absorbance after 12 h and reduction to less than 25% after 24 h. Therefore, we evaluated the cytoprotective effect of purified betanin against ROS/RNS for a short treatment time of 3–6 h, during which betanin remained mostly stable. The results showed significant cytoprotection against H_2_O_2_ toxicity even after 3 h of pretreatment (Figure 6). Both 3 h and 24 h treatments with quercetin and only the 24 h treatment with betanin induced the expression of Nrf2-regulated antioxidant genes, whereas the 3 h treatment with betanin did not induce it; this suggests that the protective effect of the 3 h betanin pre-treatment against H_2_O_2_ involved a different mechanism compared to the long-term betanin or quercetin treatment (Figure 7). The effects of betanin degradation products should be considered in the 24 h betanin treatment condition.

Betanin also showed a cytoprotective effect against the active nitrogen species ONOO^−^. As shown in Figure 2, Figure 3 and Figure 4, betanin and ONOO^−^ reacted directly at a neutral pH, and this reaction protected plasmid DNA and liposomes. These results strongly suggest that betanin in the medium removed ONOO^−^ by a direct reaction before ONOO^−^ acted on the cultured cells. In addition, a cytoprotective effect against ONOO^−^ toxicity was observed when betanin and ONOO^−^ were co-administered, suggesting that betanin directly scavenged ONOO^−^, as evident from the results of the in vitro antioxidant assay. To the best of our knowledge, this is the first study to demonstrate the cytoprotective effects of betanin against ONOO^−^ toxicity.

Our findings also suggest that the antioxidant activity of purified betanin was similar to or higher than those of ascorbic acid, tocopherol, and quercetin, which are well-known antioxidants.

Our study has the limitation that the intracellularly absorbed betanin was not quantified. In addition, the function of betanin in cells other than dermal fibroblasts needs to be confirmed.

## 4. Materials and Methods

### 4.1. Purification of Betanin

Red beetroots (*Beta vulgaris* L.) were cultivated and harvested in open-air fields by contract farmers in Eniwa-shi, Hokkaido, Japan. Betanin was extracted and purified using the following method. Red beetroots (1 kg) were squeezed using a juicer, and 0.3% (*w*/*v*) citric acid (Wako Pure Chemical Industries, Ltd., Osaka, Japan) was added to the beetroot juice to adjust the acidic conditions (pH 2.5–3.0). For deproteination, 50% (*w*/*v*) saturated ammonium sulfate (Wako Pure Chemical Industries, Ltd., Osaka, Japan) was added. After incubation for >30 min at 4 °C, centrifugation was performed for 20 min at 8000 rpm and 4 °C. The supernatant (0.6 L) was poured directly onto 200 mL of resin (COSMOSIL 75C18-OPN, Nacalai Tesque Co., Ltd., Kyoto, Japan) that had been pre-washed with methanol and placed in a Büchner funnel. After washing the resin with 1.5 L of a 0.3% citric acid solution, the selective elution of betanin with 0.6–0.7 L of a 10% EtOH-containing 0.3% citric acid solution was performed. The betanin-containing solution was evaporated under low pressure and re-dissolved in 6–7 mL of Milli-Q water (betanin concentration >10 mM). For co-precipitating betanin with citric acid, the betanin-containing solution was incubated for 1 week at 4 °C. The precipitate was filtered on filter paper, washed twice with 1 mL of 90–95% EtOH, and dried under low pressure to obtain a citric acid-free powder. The betanin concentration was calculated by measuring the absorbance at 538 nm using an ultraviolet–visible (UV–VIS) spectrophotometer (U-3310 or U-2910 Spectrophotometer, Hitachi, Ltd., Tokyo, Japan), considering the molar extinction coefficient of betanin ε_538_ = 60,000 M^−1^·cm^−1^ [23]. Commercially available betanin was purchased from Tokyo Chemical Industry Co., Ltd. (Tokyo, Japan).

### 4.2. HPLC Analysis

An HPLC system (Hitachi Elite LaChrom L-2130, manufacturing No. 18E41-032, Hitachi Ltd., Tokyo, Japan) and a reverse-phase column Puresil C_18_ (5 μm, 4.6 × 150 mm, part no. N21017, Waters, Milford, MA, USA) with a pre-column (guard-pak insert, Resolve Sillica 90A 10 μm, part no. WAT085825, Waters, Milford, MA, USA) were used to evaluate the purification of betalains. Chromatography was performed using two mobile phases, i.e., (A) 1% aqueous formic acid, (B) 80% aqueous acetonitrile (B). A linear elution gradient was used starting with 2% B and increasing to 33% B in 30 min at a flow rate of 0.8 mL min ^−1^ with an injection volume of 10 µL. The eluant was monitored at 538 nm.

### 4.3. Peroxynitrite (ONOO^−^) Synthesis

ONOO^−^ was synthesized using the method described by Patel and Darley-Usmar [24]. Ice-cold 0.6 M sodium nitrite (30 mL) and acidified 0.7 M hydrogen peroxide (in 0.6 M HCl, 30 mL) were mixed by pumping them into a Y-shaped connector. The resulting acidic ONOO^−^ was immediately quenched with 1.5 M NaOH (30 mL). Excess hydrogen peroxide was removed by adding 1.3 g of manganese dioxide (MnO_2_), which was further removed by centrifugation (4000 rpm for 5 min at 4 °C). The solution was frozen at −80 °C; ONOO^−^ tended to form a yellow top layer due to freeze fractionation, which was scraped for further studies (typical concentrations were 200–300 mM). The concentration of ONOO^−^ was determined by measuring the absorbance at 302 nm and using the molar extinction coefficient ε_302_ nm of 1670 M^−1^ cm^−1^ [24].

### 4.4. Application of the 2,2-Diphenyl-1-Picrylhydrazyl (Dpph) Assay

The antioxidant capacity of betanin was estimated using the DPPH free-radical scavenging assay [25]. Briefly, 20 µL of analytical sample with different concentrations and 80 µL of 0.1 M Tris-HCl buffer (pH 7.4) were placed into a 96-well microplate. Subsequently, 100 µL of a 0.2 mM DPPH (Wako Pure Chemical Industries, Ltd., Osaka, Japan) ethanol solution was added to the mixture. The reaction solution was kept at room temperature for 30 min in the dark. A microplate reader was used to measure the absorbance of the reaction solutions at 517 nm. A mixture of 120 µL of ethanol and 80 µL of 0.1 M Tris-HCl buffer was measured as the blank, and its absorbance was subtracted from the absorbance of all wells. The percentage DPPH scavenging was calculated from the following Equation (1):

DPPH scavenging% = [(A517 _control (DPPH)_ − A517 _antioxidant (DPPH + antioxidant)_)/A517_control (DPPH)_] × 100 (1)

To account for the background absorbance of betanin at 517 nm, a mixture of 20 µL of betanin at different concentrations and 80 µL of 0.1 M Tris-HCl buffer (pH 7.4) plus ethanol instead of the DPPH solution was used. The percentage of DPPH scavenging (betanin) was calculated from the following Equation (2):

DPPH scavenging% _(betanin)_ = [(A517 _control (DPPH)_ − A517 _betanin (DPPH + betanin)_ − A517 _betanin background (EtOH + betanin)_)/A517_control (DPPH)_] × 100 (2)

The DPPH IC_50_ and TEAC were calculated to express the antioxidant capacity of the analytical samples, based on the method described by Shimamura et al. [25]. The DPPH assay for each compound was conducted at three or four different concentrations, and it was confirmed that all measurement points were on the regression line. We also confirmed that the two points around the 50% inhibition did not deviate from the regression line, and the DPPH IC_50_ value was calculated by interpolation by connecting the two points around the 50% inhibition. TEAC was estimated as follows (3):

TEAC (µmol TE/µmol) = DPPH IC_50_ of Trolox (µmol/L)/ IC_50_ of analytical sample (µmol/L) (3)

The concentrations of quercetin (Cayman Chemical Company, Ann Arbor, MI, USA) and ascorbic acid sodium salt (Wako Pure Chemical Industries, Ltd., Osaka, Japan) were estimated using the molar extinction coefficients ε_376_ = 21.9 mM^−1^·cm^−1^ and ε_265_= 7.0 mM^−1^·cm^−1^, respectively. Betanin and ascorbic acid were dissolved in water, and quercetin, α-tocopherol (Wako Pure Chemical Industries, Ltd., Osaka, Japan), and Trolox (Cayman Chemical Company, Ann Arbor, MI, USA) were dissolved in ethanol.

### 4.5. Direct Reaction of Betanin and Quercetin with ONOO^−^

The analyzed compounds were directly reacted with ONOO^−^ to determine their ONOO^−^ scavenging activity. Betanin (10 μM) or quercetin (20 μM) was added to 0.25 M phosphate buffer (pH 7.4), and 5 μL of ONOO^−^ solutions at different concentrations was added. The total reaction mixture was 1 mL. The absorbance spectra of the reaction mixtures were measured using a UV–VIS spectrophotometer (Hitachi, Ltd., Tokyo, Japan) after 3 min of incubation. Five microliters of 0.5 M NaOH was used instead of ONOO^−^ as a control.

### 4.6. Inhibition of ONOO^−^-Dependent Plasmid DNA Cleavage

DNA cleavage was analyzed by detecting the conversion of the SC form of the pUC19 plasmid DNA (Invitrogen, Waltham, MA, USA) to the OC and linear forms. DNA (50 ng) was incubated with 0.1 mM SIN-1 (DOJINDO, Kumamoto, Japan) [26,27] in the presence or absence of betanin in 50 mM sodium phosphate buffer (pH 7.4) for 1 h at 37 °C in the dark. Because of the low water solubility of catechins, a 20% acetone solution was used for dissolving catechin. The amount of acetone added corresponded to a final concentration of 0.1% (*v*/*v*). To exclude the effect of acetone, acetone was also added to a final concentration of 0.1% to the catechin-free control and SIN-1 groups. After incubation, DNA was purified using the FastGene Gel/PCR extraction kit (Nippon Genetics Co. Ltd., Tokyo, Japan). The DNA was collected and analyzed using 1% agarose/TBE gels. After electrophoresis, the gels were stained with 0.5 μg mL^−1^ of ethidium bromide for 30 min and then photographed under UV illumination. Signal intensity was calculated using the ImageJ software [28].

### 4.7. Lipid Peroxidation Determination

The commonly used TBARS assay was employed to measure lipid peroxidation. One gram of egg lecithin (Wako Pure Chemical Industries, Ltd., Osaka, Japan) was dissolved in 10 mL of chloroform, and chloroform was removed using an evaporator; phosphate buffer (50 mM, pH 7.0) was added to reach a 5% (*w*/*v*) concentration, and the mixture was vortexed for 5 s and then stirred for 10 min using an ultrasonic homogenizer (Sonifier 250, Branson Ultrasonics, Ferguson, MO, USA) to obtain uniform liposomes.

Since the lifetime of ONOO^−^ under neutral pH conditions is only a few seconds, it is essential to agitate it quickly and evenly in the reaction solution. Therefore, the 5% (*w*/*v*) liposome solution (pH 7.0, 750 µL) was first dispensed into 1.5 mL microtubes, and droplets of the ONOO^−^ solution were attached to the wall of the microtubes without touching the liposome solution. The lipid peroxidation reaction was then initiated by agitating quickly using a vortex shaker for 5 s. After 5 min of incubation at room temperature, 0.05% butylated hydroxytoluene (BHT, Supelco Analytical, Bellfonte, PA, USA) and a thiobarbituric acid (TBA) reagent consisting of 15% (*w*/*v*) trichloroacetic acid (Wako Pure Chemical Industries, Ltd., Osaka, Japan) and 0.375% (*w*/*v*) TBA (Nacalai Tesque Co., Ltd., Kyoto, Japan) were added, and the mixture was incubated at 95 °C for 30 min, then quenched with cold water and centrifuged at 12,000 rpm for 15 min. The supernatant was centrifuged at 12,000 rpm for 15 min, and the absorbance at 532 nm was measured using a UV–VIS spectrophotometer (U-3310, Hitachi Ltd., Tokyo, Japan) to quantify the lipid peroxides.

Although TBARSs have a maximum absorption at 532 nm, a compound with an absorption at 538 nm, such as betanin, would interfere with the TBARS measurement. Therefore, to account for the sample (betanin)-derived 538 nm absorption in the TBARS measurement, the 532 nm absorbance of the sample-containing liposome suspension was used as the sample background.

The relative lipid peroxidation (%) was calculated from the following Equations (4)–(7):

TBARS (MDA (nmol)) = A532/0.1983 − 0.0663(4)

TBARS _control_ = {A532 _ONOO_^−^ _(liposome + ONOO_^−^_)_ − A532 _background (liposome)_}/0.1983 − 0.0663 (5)

TBARS _sample_ = {A532 _ONOO- (liposome + sample + ONOO_^−^_)_ − A532 _sample background (liposome + sample)_}/0.1983 − 0.0663 (6)

Relative lipid peroxidation (%) = TBARS _sample_/TBARS _control_ × 100 (7)

The TBARS IC_50_ value is the concentration of the antioxidant sample that could inhibit 1 mM ONOO^-^-induced lipid peroxidation by 50%. The TBARS value was determined from a graph by plotting relative lipid peroxidation% against sample concentration, as shown in Figure S3.

### 4.8. Cell Culture and ROS or RNS Treatments

The mouse embryonic fibroblasts NIH3T3 (Riken BioResource Research Center, Tsukuba-shi, Ibaraki, Japan) were maintained in Dulbecco’s modified Eagle medium (D-MEM, Wako Pure Chemical Industries, Ltd., Osaka, Japan) supplemented with 10% fetal bovine serum (Gibco, CA, USA) and penicillin (100 units/mL)/streptomycin (100 µg/mL) (Wako Pure Chemical Industries, Ltd., Osaka, Japan) in a humidified atmosphere with 5% CO_2_ and 95% air at 37 °C.

A stock solution of betanin (1 mM) was prepared in distilled water. After sterilization using a syringe filter (DISMIC-13CP, ADVANTEC, Tokyo, Japan), the concentration was measured prior to use. Additionally, a stock solution of quercetin (25 mM) (Cayman Chemical Company, Ann Arbor, MI, USA) was prepared in dimethyl sulfoxide (DMSO, Wako Pure Chemical Industries, Ltd., Osaka, Japan), and its concentration was measured after filter sterilization. Quercetin was added to the culture medium so that the final concentration of DMSO was 0.1% (*v/v*). NIH3T3 cells were treated with 5 µM and 25 µM betanin and quercetin for 3 h or 24 h. The medium was then replaced with fresh medium containing 0.3 mM H_2_O_2_ (Wako Pure Chemical Industries, Ltd., Osaka, Japan) or 1.5 mM the ONOO^−^ generator SIN-1 with or without betanin or quercetin. In the quantification of gene expression experiments, NIH3T3 cells were treated with betanin or quercetin for 3 h or 24 h, and the total RNA was extracted. The degradation of betanin under cell culture conditions was also determined. Purified betanin (25 μM) was incubated under the cell culture conditions described above (D-MEM medium, 5% CO_2_, 37 °C) for 24 h, its the ultraviolet–visible spectrum was determined over time using a cuvette with a 1 cm pathlength. The absorbance was also measured using betanin-free D-MEM medium as a control.

### 4.9. Cell Viability Measurement

Cell viability was measured using the 3-(4,5-dimethylthiazol-2-yl)-2,5-diphenyltetrazolium bromide (MTT) assay. Briefly, NIH3T3 cells ware seeded in 96-well plates (at a density of 3.0 or 8.0 × 10^3^ cells/well). After culturing for 24 h, the cells were treated as described in Section 4.8. After the medium was removed, the cells were incubated for 4 h in fresh medium containing MTT (0.5 mg/mL, DOJINDO, Kumamoto, Japan). After the medium was removed, the formazan crystals were dissolved in 200 µL of DMSO, and the absorbance at the wavelength of 535 nm was measured using a microplate reader (Sunrise^TM^ rainbow, Tecan, Männedorf, Switzerland). Cell viability was expressed as a percentage of that of the control, and the experiments were performed in triplicate.

### 4.10. Total Rna Extraction and Real-Time Quantitative Polymerase Chain Reaction

NIH3T3 cells were seeded into the wells of 6-well plates (at a density of 2 × 10^5^ cells/well). After culturing for 24 h, the cells were treated for 3 h or 24 h with betanin or quercetin at different concentrations. After the medium was removed, total RNA was isolated with the Isogen Ⅱ (Nippon Genetics Co. Ltd., Tokyo, Japan) reagent. Complementary DNA (cDNA) templates were generated using the PrimeScript^TM^ RT reagent kit with gDNA Eraser (Takara Bio Inc., Kusatsu, Japan). Real-time quantitative polymerase chain reaction (RT-qPCR) was performed using TB Green Premix Ex Taq (TliRNaseH Plus, Takara Bio Inc., Kusatsu, Japan) in a Thermal Cycler Dice Real Time System Ⅱ (Takara Bio Inc., Kusatsu, Japan). Glyceraldehyde 3-phosphate dehydrogenase (*GAPDH*) was employed as an endogenous control for RT-qPCR. The fold change of the mRNA expression was calculated using the standard curve method. The primer sequences used are listed in Table S1.

### 4.11. Intracellular Reactive Oxygen/Nitrogen Species Level Detection

NIH3T3 cells were seeded into the wells of 96-well plates (at a density of 3.0 or 8.0 × 10^3^ cells/well). After culturing for 24 h, the cells were treated as described in Section 4.8. After the medium was removed, the cells were washed with phosphate-buffered saline (PBS) without Ca and Mg (pH 7.4). The cells were incubated for 30 min in fresh medium containing DCFH-DA (10 µM, Sigma Aldrich, St. Louis, MO, USA) and washed twice with PBS. Finally, intracellular DCF fluorescence was detected using a BZ-9000 fluorescence microscope (Keyence, Osaka, Japan) with excitation at 470 nm and emission at 535 nm.

### 4.12. Statistical Analyses

The data are presented as mean ± standard error of the mean or standard deviation, as applicable, and were statistically analyzed using one-way analysis of variance, followed by Dunnett’s test or Tukey’s test. All statistical analyses were performed using R ver. 3.6.0. [29]. The significance level was set at *p* < 0.05.

## 5. Conclusions

In conclusion, we improved the betanin purification method and established a purification method to produce betanin powder with long-term stability. The purified betanin obtained by the improved purification method had a significantly lower isobetanin content than the commercially available betanin dyes. The antioxidant activity of purified betanin was examined using the DPPH assay, the direct ONOO^−^ reaction, ONOO^−^-dependent DNA damage, and the lipid peroxidation reaction, and the results showed that betanin possessed higher antioxidant capacity than general antioxidants such as ascorbic acid and quercetin. Furthermore, betanin showed indirect and direct cytoprotective effects against H_2_O_2_ and ONOO^−^ cytotoxicity, respectively. The isolation of highly purified betanin in this study will enable more detailed studies on its physiological functions in the future and may potentially contribute to elucidating the food functions of betanin, betanin-containing red beets, and other similar foods.

## Figures and Tables

**Table 1:** DPPH IC_50_ and Trolox equivalent antioxidant activity (TEAC) of betanin compounds and standard antioxidants measured using the 2,2-diphenyl-1-picrylhydrazyl assay.

Tested Compounds	DPPH IC_50_ (µM)	Trolox Equivalent Antioxidant Activity (TEAC; µmol TE/µmol)
Purified betanin	49.18 ± 2.12 ^a^	7.31 ± 0.42 ^e^
Commercial betanin	55.8 ± 11.6 ^a^	6.76 ± 1.61 ^e^
Quercetin	146.52 ± 6.98 ^b^	2.45 ± 0.09 ^f^
Ascorbic acid	275.18 ± 7.9 ^c^	1.30 ± 0.04 ^f^
Trolox	358.83 ± 8.2 ^d^	1 ^f^

Data are presented as mean ± standard deviation (*n* = 3). Tukey’s test was used to compare each value, and those with different superscripts are significantly different (*p* < 0.05). For DPPH IC_50_, significant differences were observed among the four groups of betanin (both purified and commercial), quercetin, ascorbic acid, and Trolox (represented by a, b, c, and d, respectively), and there was no significant difference between the two betanin groups. For the TEAC values, a significant difference was observed between groups indicated by e and f. The two betanin groups exhibited significantly higher DPPH scavenging activity than the other three antioxidants. No significant differences existed within the groups indicated by e and f.

## Data Availability

The data presented in this study are available on request from the corresponding author.

## Supplementary Materials

The following supporting information can be downloaded at: https://www.mdpi.com/article/10.3390/ijms242015411/s1.
