# Supplementary material for: Isolation of High-Purity Betanin from Red Beet and Elucidation of Its Antioxidant Activity against Peroxynitrite: An in vitro Study
Source: Int J Mol Sci. 2023 Oct 21;24(20):15411. doi: 10.3390/ijms242015411 (PMC10607038; doi:10.3390/ijms242015411)
Supplement: Supplementary file 1 [file ijms-24-15411-s001.zip › ijms-2610988-supplementary.pdf]

Article

Isolation of High-Purity Betanin from Red Beet and Elucidation of Its Antioxidant Activity against Peroxynitrite: An in vitro Study

Yasuko Sakihama [1,*], Takashi Kato [2,†], Sopanat Sawatdee [3,†], Yo Yakushi [2], Junichi Asano [2], Hiroyo Hayashi [2], Yuya Goto [2], Makoto Hashimoto [1] and Yasuyuki Hashidoko [1]

Supplementary Material

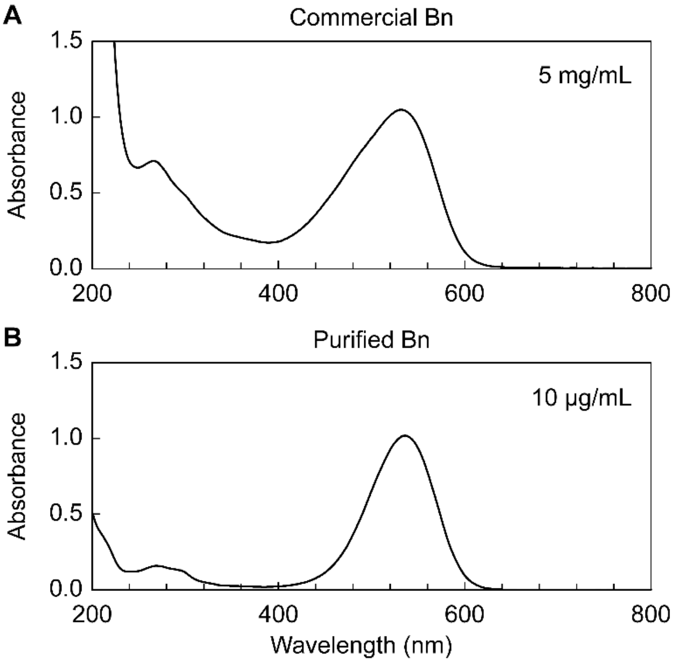

Figure S1. Ultraviolet–visible spectrum of betanin powders. (**A**) the absorption spectrum of a 5 mg/mL aqueous solution of commercial betanin powder. (**B**) the absorption spectrum of purified betanin powder 10 µg/mL aqueous solution. Ten mg of commercial betanin powder or 1 mg of purified betanin powder was dissolved in 1 mL of ultrapure water; diluted 2-fold and 100-fold, respectively; and used for spectrophotometric measurements.

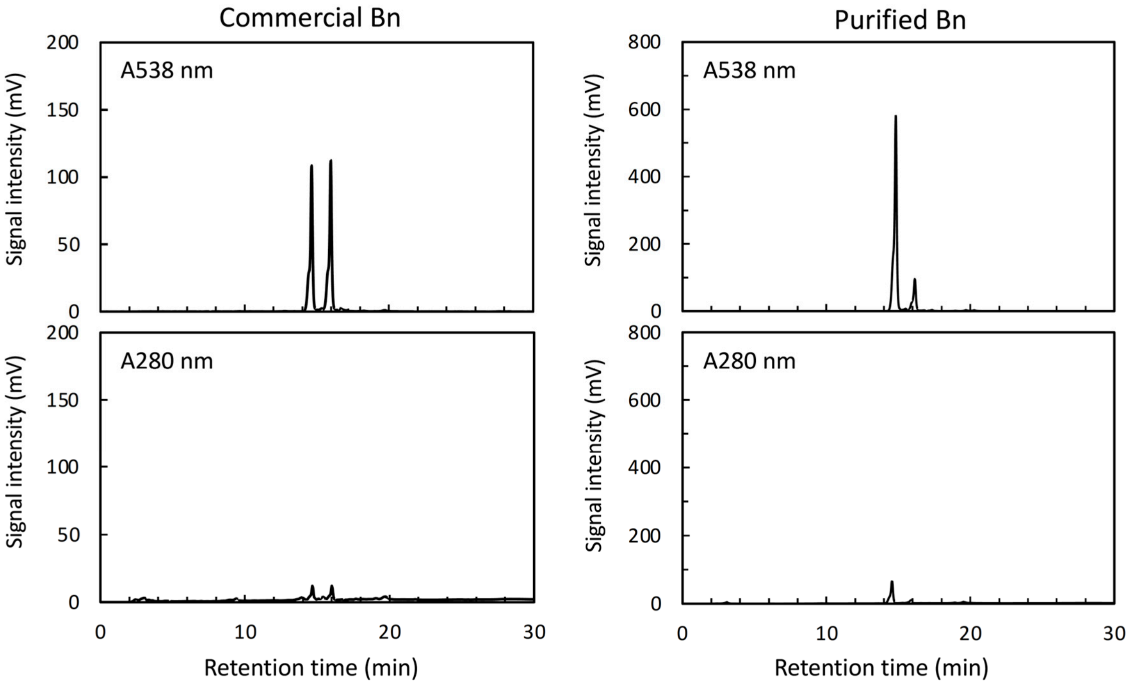

Figure S2. HPLC analysis of commercial and purified betanin.
10 μL each of (**A**) commercial betanin (65 μM) and (**B**) purified betanin (170 μM) solutions were used for analysis and monitored at 538 and 280 nm.

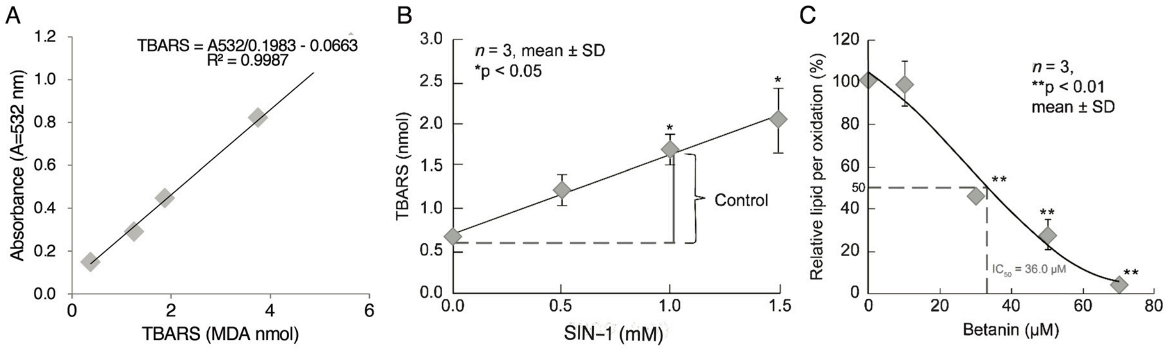

Figure S3. $ONOO^-$-dependent lipid peroxidation. (**A**) thiobarbituric acid reactive substances (TBARS) calibration curve using malondialdehyde (MDA). (**B**) TBARS formed in the presence of SIN-1 (1 mM) is used as the control (100%) in Figure S3C. (**C**) suppression of $ONOO^-$-dependent liposome oxidation by betanin (*n*=3, mean ± standard deviation).

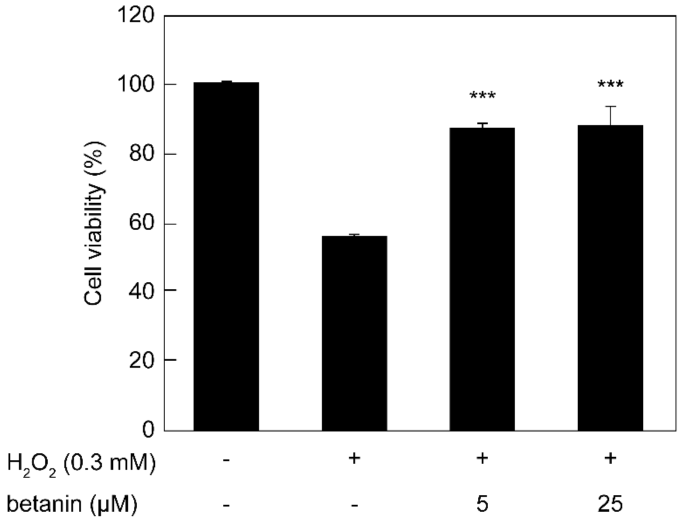

Figure S4. Cytoprotective effects of betanin against H_2O_2. Cell viability of NIH3T3 cells exposed to H_2O_2 (0.3 mM) after pre-treatment with betanin (5, 25 μM) for 24 h. Data are presented as mean ± standard error of the mean (*n*=3). Data were statistically assessed using one-way analysis of variance (ANOVA) followed by Dunnett's test. ***$p < 0.001$ compared with the H_2O_2 group

Table S1. Sequences of the primers for real-time quantitative polymerase chain reaction

Gene	Forward Sequence (5′ to 3′)	Reverse Sequence (5′ to 3′)
HO-1	ATGGCGTCACTTCGTCAGAG	GCTGATCTGGGGTTTCCCT
NQO1	GGCTGGTTTGAGAGAGTGCT	TCTGGAAAGGACCGTTGTCG
γGCS	AACACAGACCCAACCCAGAG	GTCTGGCTGAGAAGCCTTTG
GAPDH	AGAGAGGCCCTATCCCAACT	CCCTAGGCCCCTCCTGTTAT

HO-1: Heme oxygenase-1, *NQO1:* NAD(P)H quinone oxidoreductase 1, *γGCS:* γ-glutamylcysteine synthetas*e, GAPDH:* Glyceraldehyde 3-phosphate dehydrogenase
